# Mild Prenatal Stress Causes Emotional and Brain Structural Modifications in Rats of Both Sexes

**DOI:** 10.3389/fnbeh.2018.00129

**Published:** 2018-07-02

**Authors:** Carina Soares-Cunha, Bárbara Coimbra, Sónia Borges, Ana Verónica Domingues, Deolinda Silva, Nuno Sousa, Ana João Rodrigues

**Affiliations:** ^1^Life and Health Sciences Research Institute (ICVS), School of Medicine, University of Minho, Braga, Portugal; ^2^ICVS/3B’s−PT Government Associate Laboratory, Braga/Guimarães, Portugal; ^3^Clinical Academic Center-Braga (2CA), Braga, Portugal

**Keywords:** prenatal stress, anxiety, depression, BNST, hippocampus

## Abstract

Stress or high levels of glucocorticoids (GCs) during developmental periods is known to induce persistent effects in the neuroendocrine circuits that control stress response, which may underlie individuals’ increased risk for developing neuropsychiatric conditions later in life, such as anxiety or depression. We developed a rat model (Wistar han) of mild exposure to unpredictable prenatal stress (PS), which consists in a 4-h stressor administered three times per week on a random basis; stressors include strobe lights, noise and restrain. Pregnant dams subjected to this protocol present disrupted circadian corticosterone secretion and increased corticosterone secretion upon acute stress exposure. Regarding progeny, both young adult (2 months old) male and female rats present increased levels of circulating corticosterone and hyperactivity of the hypothalamus-pituitary-adrenal axis to acute stress exposure. Both sexes present anxious- and depressive-like behaviors, shown by the decreased time spent in the open arms of the elevated plus maze (EPM) and in the light side of the light-dark box (LDB), and by increased immobility time in the forced swim test, respectively. Interestingly, these results were accompanied by structural modifications of the bed nucleus of stria terminalis (BNST) and hippocampus, as well as decreased norepinephrine and dopamine levels in the BNST, and serotonin levels in the hippocampus. In summary, we characterize a new model of mild PS, and show that stressful events during pregnancy can lead to long-lasting structural and neurochemical effects in the offspring, which affect behavior in adulthood.

## Introduction

Early life adversity, including physical and emotional neglect and traumatic experiences, can induce persistent effects on physical and mental health in both animal models and humans (Heim and Nemeroff, 2001; Teicher et al., 2003). Specifically, there is now well-documented evidence that adversity in childhood increases the risk for development of personality disorders, major depression, posttraumatic stress disorder, anxiety and addictive disorders (Agid et al., 1999; Heim and Nemeroff, 2001; Dube et al., 2003; Chapman et al., 2004). The clinical importance of these findings is more evident if one considers that 80% of adults who experienced abuse or neglect early in life are predicted to suffer at least one episode of a psychiatric disorder such as depression and anxiety or a behavioral disorder such as addiction (Heim and Nemeroff, 2001; Edwards et al., 2003; Gutman and Nemeroff, 2003; McFarland et al., 2003; Espejo et al., 2007). In contrast, the predicted incidence of such disturbances is much lower in individuals abused as adults (Brown and Moran, 1994; McCauley et al., 1997), a finding that points to the existence of critical time windows during which the organism is particularly sensitive to stress-induced pathology later in life.

The stress response works through the activation of the hypothalamic-pituitary-adrenocortical (HPA) axis, that induces glucocorticoid (GC) release by the adrenals, which generate a negative feedback that requires mineralocorticoid (MR) and GC receptors (GR) in the brain (Szuran et al., 2000; Herman et al., 2016). The MR are important in the recognition of the stress and the onset of the stress response whereas GRs are activated by the presence of abnormally high levels of corticosterone and are important in the cessation of the stress reaction (de Kloet et al., 2005). Interestingly, much attention has been focused on the ability of early life adversity to program HPA activity (Tarullo and Gunnar, 2006; Heim et al., 2008). Importantly, *in utero* exposure to GC/stress has been found to be associated with long-lasting deficits in mood and affective, as well as addictive behaviors in humans (Heim and Nemeroff, 2001; Sinha, 2001; Malaspina et al., 2008) and in animal models (Oliveira et al., 2006; Mabandla et al., 2008; Markham et al., 2010; Rodrigues et al., 2012; Borges et al., 2013a,b; Soares-Cunha et al., 2014). One of the mechanisms underlying these behavioral changes may be the impact that excessive GCs reaching the fetus have on the development of brain structures involved in mood disorders. In fact, prenatal exposure to high GCs in monkeys causes a dose-dependent degeneration of hippocampal neurons, leading to reduced hippocampal volume (Uno et al., 1990). Similarly, other brain regions such as the prefrontal cortex, amygdala, bed nucleus of stria terminalis (BNST) and nucleus accumbens (NAc) are also affected in rats (Piazza and Le Moal, 1996; Murmu et al., 2006; Oliveira et al., 2006; Lupien et al., 2009; Rodrigues et al., 2012). A second mechanism that contributes to these alterations may be the induction of epigenetic alterations in specific genes. Indeed, pioneering work by Meaney and colleagues showed that the GR can be epigenetically programmed by early life adverse events in both rodents and humans (Weaver et al., 2004; Meaney et al., 2007; McGowan et al., 2009). Moreover, we have shown that prenatal GCs exposure induces long lasting epigenetic changes in dopamine receptor D2 (Rodrigues et al., 2012).

Interestingly, animal studies have also shown that male and female brain exposed to prenatal stress/GCs may undergo different reprogramming. While some argue that prenatal stress causes alterations in the HPA axis response of both male and female rats (Koehl et al., 1999), some studies point to the possibility of sex-specific alterations (Weinstock et al., 1992), thus raising doubt on the existence (or not) of a correlation between the prolonged effects of prenatal stress exposure and sex. Moreover, other studies have shown a differential impact of prenatal stress in mood according to sex (Weinstock et al., 1992; Verma et al., 2011; Weinstock, 2011).

The aim of this study was to develop a novel mild prenatal stress (PS) protocol, which consists in exposure of pregnant dams to unpredicted stress, three times per week, during the gestational days 3–20. Progeny of both sexes was characterized at adulthood (2 months old) for anxious, depressive, impulsive and reward-related behaviors. Moreover, we evaluated structural and neurochemical changes in key brain regions involved in these behavioral dimensions, namely the hippocampus, amygdala, BNST and NAc.

## Materials and Methods

### Animals

Twelve double housed virgin female Wistar han rats (8–10 weeks of age, weighing ~250 g) were housed overnight with six experienced male Wistar han rats (6 months of age, weighing ~500 g) and were maintained under standard laboratory conditions with an artificial 12-h light/dark cycle (lights on from 08:00 to 20:00), with an ambient temperature of 21 ± 1°C and a relative humidity of 50%–60%. Standard mating/pregnancy diet (4RF25, Mucedola SRL) and water were given *ad libitum*, except when stated otherwise. Males were removed from the box and females were kept individually housed from pregnancy day 0—which was considered the day when sperm was observed in the vaginal smear—forward.

Progeny from each individual litter were weaned on post-natal day 21 and pair-housed according to sex. The number of animals per litter (male and female) is indicated in Supplementary Table S1. Animals were maintained in the same housing conditions as mothers. Male and female progeny derived from at least four different litters were used for all the behavioral, physiological and neurochemical tests, to control for potential litter effects. All behavioral tests were performed when animals were 2–4 months old (61–115 days of age). All animals performed all behavioral tests.

The following behavioral tests were performed from 9:00 to 13:00: Elevated Plus Maze (EPM), Open field (OF), Light/Dark Box test (LDB) and Forced Swimming Test (FST).

The training sessions of the Variable Delay-to-Signal (VDS) and Progressive Ratio (PR) were performed from 9:00 to 19:00; one session was performed in the morning and one session was performed in the afternoon (with an interval of at least 4 h between training sessions). The test sessions of the VDS and PR were performed from 9:00 to 13:00.

The SPT was performed during the dark period, from 21:00 to 22:00.

Health monitoring was performed according to FELASA guidelines. All procedures were conducted in accordance with European Regulations (European Union Directive 2010/63/EU). Animal facilities and the people directly involved in animal experiments were certified by the Portuguese regulatory entity—Direcão-Geral de Alimentação e Veterinária (DGAV—project 023432). All protocols were approved by the Ethics Committee of ICVS.

### Prenatal Stress Protocol

From pregnancy day 3 to day 20, six randomly-selected pregnant female rats were subjected to an unpredictable stress protocol (PS, prenatal stress group) that consisted of 4 h of restraint in a cylindric box with 12 cm diameter, stroboscopic lights (Strobeyellow, Disco Pro Light) or exposure to noise (80 dB), given three times per week in a random basis. Control female rats (CTR group, *n* = 6) were left undisturbed in their home cages. At weaning day (post-natal day 21), male and female offspring were house-paired randomly, according with PS treatment (PS or CTR animals), under standard laboratory conditions: artificial 12 h light/dark cycle (lights on from 08:00 a.m. to 08:00 p.m.); room temperature 22°C; food (4RF21, Mucedola SRL) and water were provided *ad libitum*, unless stated otherwise. Animals derived from all litters were used for the experimental procedures.

#### Body Weight Monitoring

Male and female offspring body weight was monitored from birth until adulthood (from days 1 to 80 post-birth). Body weight was assessed once a week; data is presented as total body weight in grams.

### Behavioral Tests

The behavioral timeline is shown in Figure 3A.

**Figure 1:**
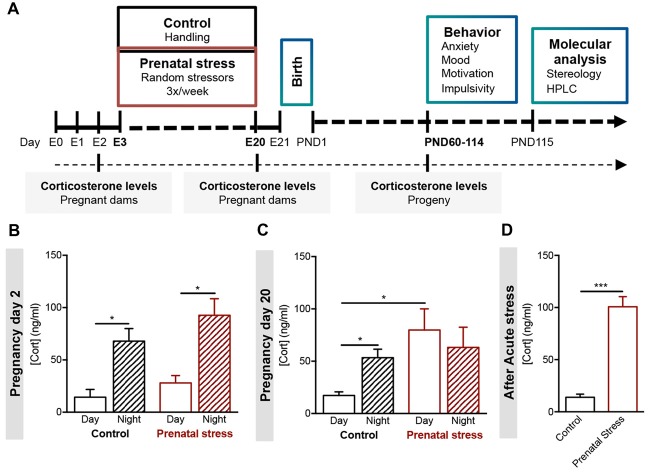
Development of a model of prenatal exposure to unpredictable mild stress. **(A)** Experimental timeline. Pregnant females Wistar han rats (age of 8 weeks, *n* = 6) were exposed to an unpredictable stress protocol (consisting of strobe lights, restraint or noise), applied three times per week in a random fashion from day 3 to day 20 of pregnancy (progeny—prenatal stress group). Progeny of control group derived from age-matched female rats that were handled daily throughout pregnancy. Blood samples were collected from all pregnant dams on day 2 and day 20, and from progeny in adulthood. Different behavioral tests were conducted in young adult male and female progeny. Stereological measurements and catecholamine levels of specific brain regions were evaluated in the progeny. **(B)** Morning (8 am) and night (8 pm) serum corticosterone levels of control and stress-exposed prenatal stress (PS) pregnant dams were measured at the beginning of pregnancy (day 2, *n* = 5) and **(C)** at the end of pregnancy (day 20, *n* = 4). PS pregnant dams present a disruption in corticosterone circadian secretion at later stages of gestation. **(D)** Corticosterone levels of control and PS mothers were also measured after exposure to an acute stressor (restraint for 30 min; *n* = 4). PS mothers present significantly higher corticosterone secretion after acute stress in comparison to controls group. Error bars denote SEM. *p < 0.05, ***p < 0.001.

**Figure 2:**
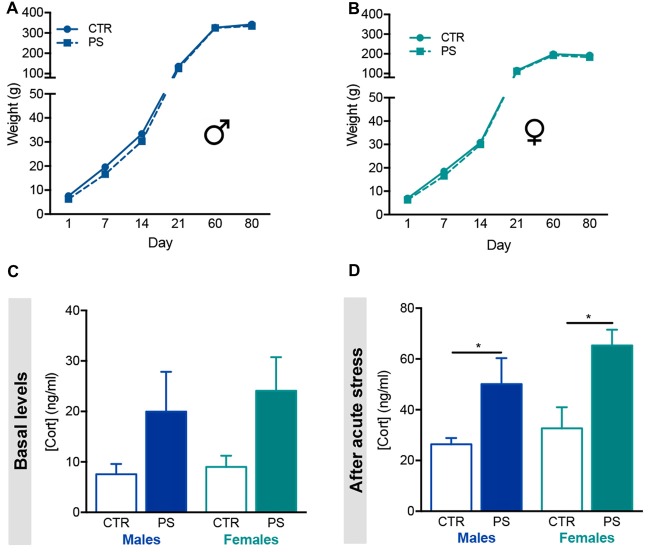
Prenatal mild stress disrupts corticosterone levels in offspring. **(A)** Body weight gain of male rats prenatally exposed to stress (PS, *n* = 28) and control group (CTR, *n* = 8), showing no differences in body weight gain as animals aged. **(B)** Body weight gain of female rats prenatally exposed to stress (PS, *n* = 20) and control group (CTR, *n* = 9), showing no differences between groups. **(C)** Basal serum corticosterone levels of male and female PS is increased in comparison with CTR rats, although not statistically significant (n_PS males_ = 6, n_CTR males_ = 6, n_PS females_ = 6, n_CTR females_ = 4). **(D)** Upon acute stress exposure (restraint for 30 min), male and female PS rats presented significantly increased serum levels of corticosterone (n_PS males_ = 8, n_CTR males_ = 4, n_PS females_ = 6, n_CTR females_ = 6). Error bars denote SEM. *p < 0.05.

**Figure 3:**
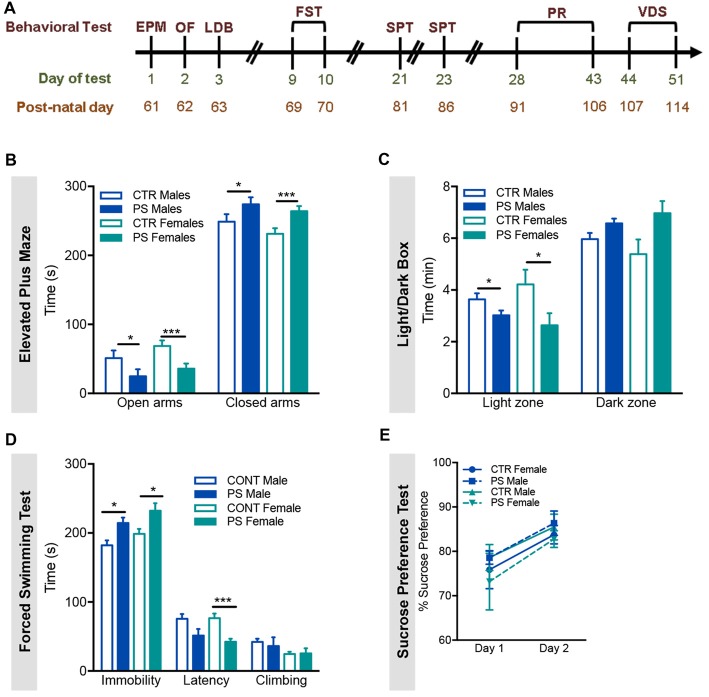
Prenatal mild stress induces anxious- and depressive-like behaviors. **(A)** Timeline of the behavioral tests performed; all rats performed the same behavioral tests in the order shown; EPM, Elevated Plus Maze; OF, Open field; LDB, Light/Dark Box; FST, Forced Swimming Test; SPT, Sucrose Preference Test; PR, Progressive Ratio; VDS, Variable Delay to Signal. **(B)** In the EPM, PS male and female rats exhibited a decrease in time spent in the open arms of the maze, when compared with control group (n_PS males_ = 9, n_CTR males_ = 9, n_PS females_ = 11, n_CTR females_ = 12), indicative of anxious behavior. **(C)** In agreement with an anxious phenotype, in the LDB test, PS male and female rats spent significantly less time in the light zone, when compared with same-sex CTR (n_PS males_ = 11, n_CTR males_ = 9, n_PS females_ = 8, n_CTR females_ = 7). **(D)** In the FST, PS male and female rats present increased immobility time and decreased latency to immobility and climbing attempts (n_PS males_ = 11, n_CTR males_ = 9, n_PS females_ = 12, n_CTR females_ = 12), a measure of behavioral despair, and suggestive of depressive-like behavior. **(E)** No major differences in SPT were found (n_PS males_ = 8, n_CTR males_ = 8, n_PS females_ = 12, n_CTR females_ = 12). This test evaluates anhedonia, another dimension of depressive-like behavior. Error bars denote SEM. *p < 0.05, ***p < 0.001.

### Anxiety Evaluation

#### Open Field (OF)

The OF test was conducted in an arena (43.2 cm × 43.2 cm) with transparent acrylic walls and white floor (Med Associates Inc., St. Albans, VT, USA). Rats were placed in the center of the arena and movement was monitored over a period of 10 min with the aid of two 16-beam infrared arrays. Total distance traveled was used as an indicator of locomotor activity.

#### Elevated Plus Maze (EPM)

The EPM test was carried out under bright white light. Animals were placed individually for 5 min in the center of a black polypropylene plus-shaped platform elevated 72.4 cm above the floor. The apparatus consisted in two open arms (50.8 cm × 10.2 cm) and two closed arms (50.8 cm × 10.2 cm × 40.6 cm; MedAssociates Inc., St. Albans, VT, USA). The number of entries into each arm and the time spent therein were recorded.

#### Light/Dark Box (LDB)

The LDB test was performed inside the OF arena (43.2 cm × 43.2 cm; MedAssociates Inc., St. Albans, VT, USA). A dark compartment was attached to one side with an opening facing the center of the OF. Animals were individually placed in the center of the illuminated part. The distance traveled and time spent in each compartment was recorded in a single trial of 10 min.

### Depressive-Like Behavior Evaluation

#### Forced Swimming Test (FST)

Rats were placed in cylinders filled with warmed water. After a 5-min pre-test session, animals were retested 24 h later (5 min test). At the end of each session, animals were placed on a heating pad before returned to their home cage. A video camera placed in front of the cylinder was used to record sessions, and later scored by an investigator blind to the experimental details. Time of immobility (passiveness; defined as time spent either immobile or making righting movements to stay afloat), latency to immobility and number of climbing attempts were scored.

#### Sucrose Preference Test (SPT)

Anhedonia was assessed by the SPT, at two time points, with a 2-day interval. Before each trial, rats were food and water deprived for 12 h. For testing, two pre-weighed bottles containing water or a 2% (m/v) sucrose solution were presented to individually housed animals for 1 h. Sucrose preference (SP) was calculated according to the formula: SP = (sucrose intake/(sucrose intake + water intake)) × 100. Anhedonia was defined as a reduction in SP relative to baseline levels.

### Impulsivity Evaluation

#### Variable Delay-to-Signal (VDS)

The VDS provides rapid and simultaneous assessment of response and decision impulsivity in rodents. This test consists of a series of trials, in a single 30 min session, in which the time period where an action (nose poke) triggers the delivery of a sugared reward is signaled by a light, presented after a variable delay (Leite-Almeida et al., 2013). Animals were kept food-deprived during the whole protocol, which included habituation, training and testing phases. The protocol was performed in a nearly square shaped (25 cm × 25 cm) 5-hole operant chamber (OC; TSE Systems GmbH, Germany). Five square apertures (2.5 × 2.5 cm; #1–#5) with a 3W light bulb and infrared photo beams to detect movements were distributed in a slightly curved wall. On the opposite wall there was another aperture (#6), also equipped with light and photo beams, connected to a food dispenser. Above aperture #6 a house light illuminating the operant chamber. Four chambers were simultaneously used each placed inside sound attenuating chambers, with electrical fans providing ventilation and white noise.

In short, animals were habituated for 2 days. Afterwards, animals were exposed to 10 training sessions that occurred twice daily, with a 5 h interval in between, for five consecutive days. Each *training* session was initiated by turning on the house light and delivering one sugared pellet in the food magazine, the collection of which started an intertrial interval (ITI) of 3 s. Trials then started, consisting of a 3 s period with only the house light on (delay period), followed by lightning of the response aperture for 60 s (response period). Nose pokes in this aperture were either punished with a timeout period in complete darkness, if performed during the delay period (premature responses), or rewarded with the delivery of a pellet if performed during the response period. Collection of a food reward always triggered a 3 s ITI, before a new trial begun. Training sessions were carried until 100 trials were completed or until 30 min had elapsed, on several consecutive days until average premature responding stabilized. Animals failing to complete 100 trials in the 30 min limit at the end of the training period were removed from the task.

At the end of the training period animals were exposed to the single VDS testing day. The VDS testing session occurred on a single day and consisted of 120 trials, similar to those previously described, with the exception of the delay, which was 3 s in the first and the last 25 trials and randomly either 6 or 12 s in the middle 70 trials (leading to a 3 s—6/12 s—3 s configuration).

### Motivation Test

#### Progressive Ratio (PR) Schedule of Reinforcement

The PR test is a direct reflection of motivation, since it determines the amount of work (breakpoint) that rats are willing to exert to obtain food rewards in an operant task. The protocol was performed according to previous descriptions (Wanat et al., 2010; Soares-Cunha et al., 2016). Rats were placed and maintained on food restriction (~7 g per day of standard lab chow) to maintain 90% free-feeding weight. Forty-five milligram food pellets (F0021; BioServ), used in the behavioral protocol, were placed in their home cages on the day before the first training session to familiarize the rats with the food pellets. Behavioral sessions were performed in operant chambers (Med Associates).

In short, animals were exposed to 6 days of continuous reinforcement (CRF) training, in which each lever pressing resulted in the delivery of one food pellet, followed by one session of fixed-ratio (FR) 1 (in which one lever pressing is required to obtain one food pellet), four sessions of FR 4 (in which four lever presses are required to obtain one food pellet) and one session of FR8 (in which eight lever presses are required to obtain one food pellet). On the test day, rats were exposed to PR test. PR sessions were identical to FR4 sessions except that the operant requirement on each trial (T) was the integer (rounded down) of 1.4^(T-1)^ lever presses, starting at one lever press. PR sessions ended after 15 min elapsed without completion of the response requirement in a trial.

### Corticosterone Measurement

Mothers’ corticosterone levels were determined in blood samples (250 μl) withdrawn from the tail vein before the beginning of the stress protocol, at two distinct time-points (at the beginning of the light cycle (8:00) and at the beginning of the dark cycle (20:00)), and after the last day of stress protocol (at the beginning of the light cycle (8:00) and at the beginning of the dark cycle (20:00)). Adult progeny’s (male and female CTR and PS animals derived from at least four different litters) corticosterone levels were also determined in blood samples (250 μl) withdrawn from the tail vein before a 30 min restraint stress protocol, and 120 min afterward. Corticosterone concentration was determined using an ELISA corticosterone kit (ENZO Life Sciences) according to the manufacturers guidelines. Briefly, corticosterone from 10 μl of plasma was extracted and ran in the corticosterone assay that relies on the recognition of corticosterone from a specific ELISA antibody. Quantification of corticosterone from samples was calculated as the average net optical density bound for each standard and sample.

### Corticosterone Levels in Response to Acute Stress

To assess the response of the HPA axis to acute stress, blood from the tail (tail pinching) of the animals was collected at 14:00 (for assessment of corticosterone basal levels). Immediately after this period animals were exposed to an acute stress, consisting of a 30 min restraint protocol. Rats were individually placed in a plastic cylindrical box with holes. Two hours after stress blood was again collected by tail pinching.

Pregnant stress-exposed and control dams were exposed to the corticosterone response to stress assay on gestation day 20.

Progeny (male and female CTR and PS) corticosterone response to stress was assessed on post-natal day 60.

Corticosterone levels were measured in the plasma of rats as described above.

### Macrodissection

Male and female CTR and PS animals (*n* = 6/group, derived from at least four different litters) were deeply anesthetized with lethal dose of pentobarbital, decapitated and heads were immediately snap-frozen in liquid nitrogen. Brain areas of interest were rapidly dissected on ice under a stereomicroscope, observing anatomical landmarks (according to Paxinos and Watson, 2005). Samples were snap-frozen (dry ice) and stored at −80°C until use.

### Neurochemical Evaluation

Levels of catecholamines were assayed by high-performance liquid chromatography, combined with electrochemical detection (HPLC/EC) using a Gilson instrument (Gilson, Middleton, WI, USA), fitted with an analytical column (Supleco Supelcosil LC-18 3 mM, Bellefonte, PA, USA; flow rate: 1.0 ml min^−1^). Samples were stored overnight in 0.2 N perchloric acid at −20°C, sonicated (5 min on ice) and centrifuged at 5000 *g*. The resulting supernatant was filtered through a Spin-X HPLC column (Costar, Lowell, MA, USA) to remove debris and 150 ml aliquots were injected into the HPLC system, using a mobile phase of 0.7 M aqueous potassium phosphate (pH 3.0) in 10% methanol, 1-heptanesulfonic acid (222 mg l^−1^) and Na-EDTA (40 mg l^−1^). A standard curve using known concentrations of all catecholamines was run each day.

### Histological Procedures

Male and female CTR and PS animals (*n* = 4/group, derived from at least four different litters) were anesthetized with pentobarbital and were transcardially perfused with 0.9% saline followed by 4% paraformaldehyde (PFA). Brains were removed and placed in 4% PFA. After 4 weeks in PFA, brains were split into two hemispheres by a midsagittal section and processed for stereology, according to the procedure previously described (Keuker et al., 2001). Briefly, they were included in glycolmethacrylate (Tecnovit 7100) and every other microtome-cut section (30 μm) was then collected on a gelatinized slide, stained with Giemsa, and mounted with Entellan New (Merck). The shrinkage factor was calculated according to Madeira et al. (1990).

### Stereological Procedures

Volume estimations was obtained using StereoInvestigator^®^ software (MicroBrightField, Williston, VT, USA) and a motorized microscope (Axioplan 2, Carl Zeiss, Hamburg, Germany) attached to a camera (DXC-390, Sony Corporation, Tokyo, Japan). The Cavalieri’s principle (Gundersen et al., 1999) was applied to evaluate the volume of each region. For BNST and amygdala sub-regions, central amygdala (CeA) and basolateral amygdala (BLA; brain regions identified using a brain atlas (Paxinos and Watson, 2005)), every 4th section was used. For NAc sub-regions, NAc core and NAc shell (brain regions identified using a brain atlas (Paxinos and Watson, 2005)), every eighth section was used. For hippocampal sub-regions, *cornus ammonis* (CA) 1, CA3 and dentate gyrus (DG; brain regions identified using a brain atlas (Paxinos and Watson, 2005)), every 12th section was used. The volume of the region of interest was calculated from the number of points that fell within its boundaries and the distance between the systematically sampled sections.

Total cell numbers were estimated using the optical fractionator method (West et al., 1991). Briefly, a grid of virtual 3D-boxes (30 μm × 30 μm × 15 μm) equally spaced (using the same grid spacing as for volume estimations) was superimposed on every section of the lamina of interest and cells within boxes were counted. Coefficients of error were automatically computed, according to the formulas of Gundersen et al. (1999) for cell numbers and Gundersen and Jensen (1987) for volume estimations. Glial cells were not included in the estimations, and the discrimination between neuronal and glial cell body profiles was based on the criteria described before (Ling et al., 1973; Peinado et al., 1997).

### Statistical Analysis

Normality tests were performed for all data (Shapiro-Wilk test). Outliers were identified using Tukey’s fences; outliers were removed before proceeding with statistical analysis.

Student’s *t*-test was used for analysis of corticosterone levels; stereology data (volume and number of cells); catecholamine levels and the behavioral tests, with the exception of VDS (experimental group × delay) and CRF and FR trainings of the PR test (experimental group × day of training), in which analysis of variance (ANOVA) and Bonferroni *post hoc* test were used.

Since catecholamine levels within the BNST distribution was not normal, non-parametric Mann-Whitney test was applied.

Results are presented as mean ± SEM. All statistical analysis was performed using Prism GraphPad (v7) and results were considered significant for *P* ≤ 0.05.

## Results

### Establishment of a Maternal Chronic Unpredictable Mild Stress Protocol

We developed a model of maternal exposure to chronic unpredictable mild stress, which consisted in exposure to three distinct stressors—loud noise, strobe lights and restraint—with the duration of 4 h per day, three times per week, on a random order. The protocol began on pregnancy day 3 and extended until day 20 of pregnancy (Figure 1A).

We were able to observe significant changes in circulating corticosterone levels measured in the plasma of mothers exposed to gestational stress (PS). Before the beginning of the stress protocol, pregnant dams showed normal levels of corticosterone, with a low peak in the morning blood collection and a high peak in the night collection, as expected (Figure 1B; CTR: *t*_(6)_ = 3.81, *p* = 0.009; PS: *t*_(5)_= 4.1, *p* = 0.009). At the end of stress protocol, the circadian rhythm of corticosterone secretion was disrupted (Figure 1C; CTR: *t*_(8)_ = 4.1, *p* = 0.003; PS: *t*_(6)_ = 0.6, *p* = 0.593; CTR day vs. PS day: *t*_(7)_ = 3.4, *p* = 0.0109). In addition, we also found that PS-exposed mothers presented increased release of corticosterone upon exposure to an acute stress (Figure 1D; *t*_(6)_ = 8.5, *p* < 0.001), suggesting impairment in the negative feedback of the HPA axis.

### Prenatal Exposure to Unpredictable Mild Stress Enhances Corticosterone Release in the Progeny

We evaluated the body weight gain of both male and female control (CTR) rats and those prenatally exposed to PS. There were no differences in the weight gain of the progeny since birth until 10 weeks of age (Figures 2A,B). We found a trend for increased corticosterone in both sexes of PS group, though it was highly variable between animals (Figure 2C; males: *t*_(9)_ = 1.8, *p* = 0.103; females: *t*_(8)_ = 1.8, *p* = 0.1143). Interestingly, both PS males and PS females secreted higher levels of corticosterone in response to an acute stress (Figure 2D; males: *t*_(10)_ = 3.1, *p* = 0.01: females: *t*_(10)_ = 3.1, *p* = 0.01), suggesting impairment in the HPA axis activity caused by PS exposure.

### Prenatal Exposure to Mild Stress Causes Anxious-Like Behavior in Both Sexes

In order to assess if PS exposure could have an impact in the behavior of adult offspring, we exposed 2-month old rats to the EPM, the OF and the LDB test to assess anxious behavior. In the EPM, both male and female PS animals spent significantly less time in the open arms (Figure 3B; males: *t*_(15)_ = 2.2, *p* = 0.04; females: *t*_(20)_ = 3.9, *p* = 0.0008), suggesting increased anxious behavior. In agreement, in the LDB test, PS exposure caused a significant decrease in time spent in the light side of the box (anxiogenic) in comparison with CTR rats in both sexes (Figure 3C; males: *t*_(13)_ = 2.1, *p* = 0.05; females: *t*_(13)_ = 2.17, *p* = 0.049). Regarding the OF test, no significant differences were found in the distance traveled (Supplementary Figure S1A) or time spent (Supplementary Figure S1B) in the center and the periphery of the arena between PS and CTR rats of both sexes.

### Prenatal Exposure to Mild Stress Causes Depressive-Like Behaviors in Both Sexes

Next, we also evaluated depressive-like behavior using the FST. PS exposure causes a significant increase in the time rats spend immobile in both sexes (Figure 3D; males: *t*_(14)_ = 3.1, *p* = 0.0087; females: *t*_(22)_ = 2.56, *p* = 0.0178), consistent with increased depressive-like behavior. In addition, we examined the hedonic status of PS-exposed rats by performing the SPT. Interestingly, no major differences were observed in the SPT test between groups (Figure 3E).

### Prenatal Exposure to Mild Stress Does Not Induce Motivational Deficits Nor Causes Impulsivity Traits

Next, we evaluated the motivational status of animals using the PR schedule of reinforcement (PR test), which measures the willingness of the animals to work to obtain a food pellet (reward). The breakpoint, i.e., when animals give up, represents a direct measure of motivation. First, we observed that both male and female animals acquired instrumental conditioning in the CRF training, shown by the increase in lever press over the days of training (Supplementary Figure S2A; CONT males vs. PS males: *F*_(4, 28)_ = 109, *p* < 0.001; CONT females vs. PS females: *F*_(4,40)_ = 91.4, *p* < 0.001). Similarly, both sexes increased lever pressing over fixed ratio (FR) days of training (Supplementary Figure S2B; CONT males vs. PS males: *F*_(4,120)_ = 50.7, *p* < 0.001; CONT females vs. PS females: *F*_(4,160)_ = 45.1, *p* < 0.001), and show significantly more lever pressing in the active lever than in the inactive lever (Supplementary Figure S2B; CONT males vs. PS males: *F*_(3,30)_ = 136.4, *p* < 0.001; CONT females vs. PS females: *F*_(3,40)_ = 120.8, *p* < 0.001). These results indicate that PS rats of both sexes are able to learn the task in a similar manner. No significant differences were observed in the breakpoint of PS animals in comparison with CTR rats in both sexes, suggesting that PS did not influence motivation (Supplementary Figure S2C; males: *t*_(15)_ = 0.6, *p* = 0.531; females: *t*_(20)_ = 1.0, *p* = 0.318). Similarly, no differences in the number of food pellets consumed during the PR session were observed (Supplementary Figure S2D).

We also tested animals in the VDS test, a paradigm designed to assess impulsivity. Interestingly, no significant differences were found in the VDS test (Supplementary Figure S2E).

### Structural Changes in the Limbic System Caused by Prenatal Stress Exposure

In order to better understand the cause of the behavioral deficits observed in PS rats, we measured the volume and the number of cells, and evaluated catecholamine content of different regions of the limbic system, namely the BNST, hippocampus, amygdala and NAc.

**Figure 4:**
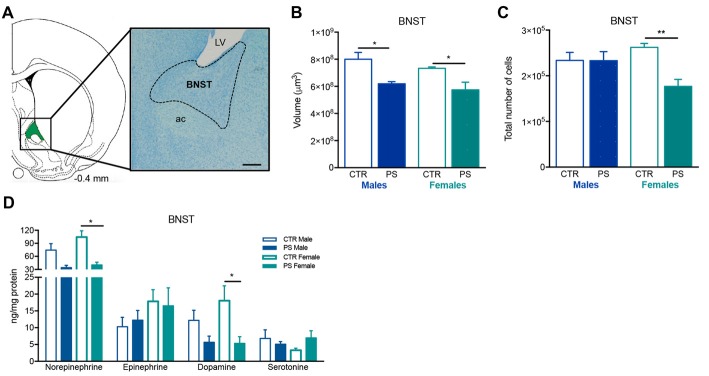
Exposure to prenatal mild stress alters the structure and neurochemical content of the bed nucleus of stria terminalis (BNST). **(A)** Representative image of the BNST anatomy, using a methacrylate coronal section of a rat’s brain; numbers represent distance to Bregma; scale bar = 1 mm. **(B)** PS animals of both genders show a significant reduction in the volume of the BNST (n_PS males_ = 7, n_CTR males_ = 4, n_PS females_ = 4, n_CTR females_ = 4). **(C)** Females also present a decrease in the total number of cells (n_PS males_ = 4, n_CTR males_ = 3, n_PS females_ = 3, n_CTR females_ = 4), whereas males present a similar number as CTR. **(D)** HPLC measurement of catecholamines revealed that the BNST of female PS animals present a reduction in norepinephrine and dopamine levels in comparison with CTR female rats, and there is a trend for decreased levels of the same amines in male PS rats; no major differences were found in other neurotransmitter levels (n_PS males_ = 4, n_CTR males_ = 4, n_PS females_ = 4, n_CTR females_ = 4). Error bars denote SEM. *p < 0.05, **p < 0.01.

PS causes a significant decrease in the volume of the BSNT in both males and females (Figures 4A,B; males: *t*_(9)_ = 4.3, *p* = 0.0018; females: *t*_(6)_ = 2.8, *p* = 0.0322). Females also presented a significant reduction in total number of cells in the same brain region (Figure 4C; *t*_(5)_ = 4.4, *p* = 0.0071). Additionally, PS exposure caused a trend for decreased levels of norepinephrine and dopamine of male PS rats (Figure 4D; norepinephrine: *U*_6_ = 2.0, *p* = 0.114; dopamine: *U*_6_ = 2.0, *p* = 0.057), and a significant decrease in PS female rats in comparison to CTR female rats (Figure 4D; norepinephrine: *U*_6_ = 0.0, *p* = 0.0286; dopamine: *t*_(6)_ = 2.6, *p* = 0.039).

**Figure 5:**
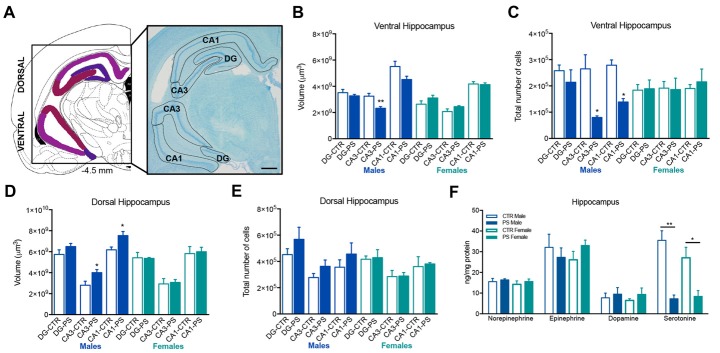
Exposure to prenatal mild stress alters the structure and neurochemical content of the hippocampus. **(A)** Representative image of the ventral and dorsal hippocampus anatomy, using a methacrylate coronal section of a rat’s brain; numbers represent distance to Bregma; scale bar = 1 mm. **(B)** Male PS animals showed a significant reduction in the volume of the CA3 sub-region of the ventral hippocampus, with no significant differences in other sub-divisions of this brain region in comparison with male CTR rats; female PS rats did not show any significant difference in comparison with CTR (rats n_PS males_ = 5, n_CTR males_ = 4, n_PS females_ = 5, n_CTR females_ = 4). **(C)** Male PS rats also presented a reduced number of cells in CA1 and CA3 sub-regions of the ventral hippocampus, but not PS females (rats n_PS males_ = 4, n_CTR males_ = 4, n_PS females_ = 4, n_CTR females_ = 3). **(D)** Male PS animals showed a significant increase in the volume of the CA1 and CA3 sub-regions of the dorsal hippocampus, with no significant differences in dentate gyrus (DG) in comparison with male CTR rats; female PS rats did not show any significant difference in comparison with CTR female rats (n_PS males_ = 6, n_CTR males_ = 4, n_PS females_ = 5, n_CTR females_ = 4). **(E)** No differences were observed in the total number of cells between PS and CTR groups in the dorsal hippocampus (n_PS males_ = 6, n_CTR males_ = 4, n_PS females_ = 5, n_CTR females_ = 4). **(F)** Hippocampal catecholamine profile showing a reduction in serotonin levels in both male and female PS groups in comparison with same-sex CTR rats; no major differences were found in other neurotransmitters (rats n_PS males_ = 4, n_CTR males_ = 4, n_PS females_ = 4, n_CTR females_ = 4). Error bars denote SEM. *p < 0.05, **p < 0.01.

A significant decrease in the volume and total number of cells of the CA3 layer of the ventral hippocampus (Figures 5A,B, volume: *t*_(7)_ = 3.4, *p* = 0.0111; Figure 5C, number of cells: *t*_(6)_ = 3.4, *p* = 0.0141) and in the total number of cells in the CA1 sub-region (Figure 5C; *t*_(6)_ = 5.9, *p* = 0.0011) in PS males, but not in females. On the other hand, CA3 and CA1 sub-regions of the dorsal hippocampus were significantly enlarged in male PS rats in comparison with male CTR rats (Figure 5D; dorsal CA1: *t*_(8)_ = 2.6, *p* = 0.031; CA3: *t*_(6)_ = 2.6, *p* = 0.030), with no differences in the total number of cells (Figure 5E). No differences were found in females. Both male and female PS animals presented a significant decrease in the levels of serotonin in the hippocampus, when compared with same-sex controls (Figure 5F; males: *t*_(6)_ = 5.7, *p* = 0.001; females: *t*_(6)_ = 3.2, *p* = 0.019).

**Figure 6:**
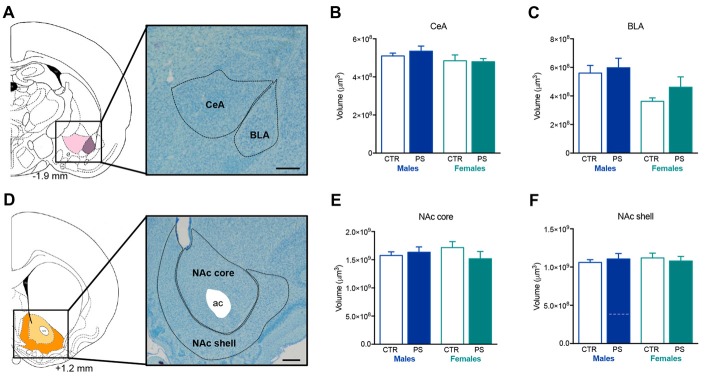
PS exposure does not alter the volume of the amygdala or the nucleus accumbens (NAc). **(A)** Representative image of the amygdala sub-regions, centralamygdala (CeA) and basolateral amygdala (BLA), using a methacrylate coronal section of a rat’s brain; numbers represent distance to Bregma; scale bar = 1 mm. PS exposure does not alter the volume of **(B)** CeA or **(C)** BLA of both male and female PS rats in comparison with same-sex controls (rats n_PS males_ = 6, n_CTR males_ = 4, n_PS females_ = 5, n_CTR females_ = 3). **(D)** Representative image of the NAc sub-regions, NAc core and NAc shell, using a methacrylate coronal section of a rat’s brain; numbers represent distance to Bregma; scale bar = 1 mm. PS exposure does not alter the volume of **(E)** NAc core or **(F)** NAc shell of both male and female PS rats in comparison with same-sex CTR (rats n_PS males_ = 9, n_CTR males_ = 4, n_PS females_ = 5, n_CTR females_ = 3). Error bars denote SEM.

No differences were found in the volume of the amygdala sub-regions (Figure 6A)—CeA (Figure 6B) and BLA (Figure 6C)—and the NAc (Figure 6D)—core (Figure 6E) and shell (Figure 6F) sub-regions.

## Discussion

Since early studies by David J. Barker focusing on the *fetal and developmental origins of adult disease*, a large effort has been made to identify how early life adversity can program adult life. In humans, the large proportion of information about the long-term effects of stress exposure during pregnancy on the offspring of human subjects has been obtained from retrospective studies (Brouwers et al., 2001; Weinstock, 2001; Kofman, 2002; Gutteling et al., 2006). These studies have been helpful to associate maternal stress with impaired metabolic, immune and neuropsychological outcomes in the progeny (Lupien et al., 2009). However, one challenge of human studies is that external variables are not easily controlled, and it is very difficult to evaluate the long-term consequences of PS without interference of daily life stress. In this perspective, the development and study of animal models of PS, in which the timing, duration and intensity of exposure to stress is strictly controlled, poses a great advantage for this type of studies.

In this work, we show that exposure to chronic unpredictable mild stress during pregnancy induces depressive and anxious symptoms in the male and female adult offspring, accompanied by structural and molecular changes in the hippocampus and BNST brain regions. Importantly, these findings corroborate the premise that adverse events occurring during prenatal period can enhance vulnerability for anxious and depressive phenotypes in adulthood both in animal models and humans (nicely reviewed by (Lupien et al., 2009) and (Weinstock, 2016)). Indeed, in humans, it is well recognized that the offspring of women exposed to stressors during gestation, like natural disasters or adverse life events have a higher risk of psychopathology (Weinstock, 2008; Charil et al., 2010), including hyperanxiety and depression (Van den Bergh et al., 2008; Van Lieshout and Boylan, 2010). Animal studies, in general, mimic these findings; nonetheless there are a large number of models of PS, due to the possibility of controlling stressor intensity and duration. Some works report the use of severe PS paradigms such as electric shocks daily throughout pregnancy (Estanislau and Morato, 2005; Yang et al., 2006). A large number of studies use a single stressor throughout pregnancy that often consists of restraint or bright lights (McCormick et al., 1995; Fujioka et al., 2001; Zuena et al., 2008; Lui et al., 2011; Laloux et al., 2012; Van den Hove et al., 2013). Akin to our study, others prefer the use of different stressors in a random manner, such as protocols of gestational unpredictable mild stress, to reduce the likelihood of habituation to stress (Hougaard et al., 2005; Bourke et al., 2013; Wilson et al., 2013; Wang et al., 2015a,b). Regardless of the type of PS, one consistent finding is the appearance of anxiety and depressive-like behavior in offspring (Estanislau and Morato, 2005; Zuena et al., 2008; Laloux et al., 2012; Wilson et al., 2013; Wang et al., 2015a,b).

One important consideration is that most of the studies apply stressors from day 10 of pregnancy onwards to prevent abortion, but we decided to start stress protocol at day 3. This is an important point because there are different windows of susceptibility to stress effects. For example, it has been shown that the same stressor applied in different stages of pregnancy induces different behavioral outcomes (Liu et al., 2008; Zuena et al., 2008; Fujita et al., 2010; Jia et al., 2010; Głombik et al., 2015).

One important finding is that though we observe anxious and depressive-behaviors, this PS paradigm does not affect other behavioral dimensions such as impulsivity or motivational drive, contrary to other stressors or prenatal GC exposure (Virgolini et al., 2008; Soares-Cunha et al., 2014; Weston et al., 2014). It is important to refer that in this work, animals were tested during the light period of the cycle, which may be a confounding factor in the interpretation of the behavioral data. Though several other studies also evaluate (stress effects in) behavior during the inactive period (Beeler et al., 2006; Roque et al., 2011; Borges et al., 2013b; Alves et al., 2017; Morais et al., 2017), these animals should be tested during their active period as well, which can increase the discriminatory effect of the behavioral tests (Hossain et al., 2004).

Our model of PS caused significant impairment in the production and release of corticosterone in the mothers. Although GCs are necessary in late gestation to promote fetal development (Roberts and Dalziel, 2006; Brownfoot et al., 2013; Moisiadis and Matthews, 2014a, b), inadequate levels of these hormones, such as for example when released early in pregnancy or in high levels, may disrupt the negative feedback of HPA axis, leading to a disturbance in the ability to control the release of these hormones (Roberts and Dalziel, 2006; Brownfoot et al., 2013; Moisiadis and Matthews, 2014a, b). In line with this, we show that, although basal corticosterone levels of PS progeny are similar to control animals, when exposed to an acute stress, these animals present a significant increase in corticosterone levels (in both sexes). This suggests that there is an impairment in the HPA axis, a phenomenon that has been observed in other models of PS (Levine, 1967; Hougaard et al., 2005; Weinstock, 2005; Wilson et al., 2013; Jafari et al., 2017).

Dysregulation of the HPA axis has been associated with different neuropsychiatric conditions such as anxiety and depression in humans (Varghese and Brown, 2001; Wardenaar et al., 2011) and animal models (Varghese and Brown, 2001; Wardenaar et al., 2011). In line with this view, we show that PS animals of both sexes present anxious behavior in two behavioral paradigms. Interestingly, in humans, self-reporting data suggests that PS may also induce a chronic anxiety state in adulthood (Ward, 1991; Van den Bergh and Marcoen, 2004). The anxiogenic phenotype may be caused by the volumetric alterations in the BNST observed in PS progeny, since this brain region has been linked for long to anxiety (Walker et al., 2003; Waddell et al., 2006). Besides the atrophy, the observed catecholaminergic changes may also contribute for the anxious behavior. In fact, other studies have associated an atrophy of the BNST and catecholamine deregulation with anxious behavior (Oliveira et al., 2012). In accordance, several other studies have described a unique role for this brain region in anxiety-like responses in rodents (Walker et al., 2003; Davis et al., 2010).

In addition to the notorious anxious phenotype, PS animals also revealed a significant depressive-like behavior, akin to other animal models of early life stress exposure (Borges et al., 2013a; Palacios-García et al., 2015). One potential explanation for this behavioral trait is the observed decrease in hippocampal serotonin levels of both males and females, given that depression has been strongly linked to impairments in serotonergic neurotransmission (Mahar et al., 2014). In addition to the neurochemical modifications caused by exposure to PS, substantial alterations were also observed in CA1 and CA3 hippocampal structure, being ventral hippocampus hypotrophic and dorsal hippocampus hypertrophic in PS-exposed males. Consistent with our data, other study has reported that PS causes dendritic atrophy of the pyramidal neurons of the CA3 sub-region in offspring (Jia et al., 2010). In addition, our data are in accordance with human studies that show that hippocampal atrophy is associated with recurrent depressive illness (Videbech and Ravnkilde, 2004; McKinnon et al., 2009). Despite this, it is still unclear why PS females present the same behavioral trait without evident changes in hippocampal structure. One cannot rule out that, although structurally intact, the hippocampus may be malfunctioning in the female brain, which is in agreement with the observed serotonergic neurotransmission impairment in these animals.

In addition, one cannot neglect the contribution of other brain regions for depressive behavior such as the amygdala and the NAc. Consistent with the data that we report here showing no volumetric changes in the amygdala of neither PS males nor females, others have also shown the same in depressive human patients (von Gunten et al., 2000; Hickie et al., 2007). However, functional MRI studies show that BLA is hyperactive in several mood disorders, with no apparent volumetric differences (Drevets et al., 1992; Sheline et al., 1996), so one cannot rule out the possibility of this brain region being dysfunctional and (partially) contribute to the observed phenotype. Regarding the NAc, several studies have associated stress-induced changes of this brain region with depressive behavior (Martínez-Téllez et al., 2009; Morales-Medina et al., 2009; Rodrigues et al., 2012; Bessa et al., 2013; Russo and Nestler, 2013; Haim et al., 2014; Francis et al., 2015). However, we found no structural differences in the NAc in this model of PS. Human studies also showed no changes in NAc volume in major depression (Bremner et al., 2000; Hannestad et al., 2006). We also did not find any motivational deficits in PS animals, another core symptom of depressive behavior, nor found any differences in impulsivity which has also been linked to this brain region, suggesting relatively intact NAc (Cardinal et al., 2001; Basar et al., 2010; Soares-Cunha et al., 2016). Yet, functional studies such as *in vivo* electrophysiology or *in vivo* calcium imaging in freely moving animals would be crucial to understand if this brain region (or others) is affected (or not) in this model.

Although we have analyzed the effect of early life exposure to stress in males and females, we could not observe significant differences between sexes. Despite some structural differences of the hippocampus that were observed only in males, both sexes seem to be similarly affected by PS. Curiously, other studies have reported differences between sexes (Roussel et al., 2005; Weinstock, 2007; Boersma and Tamashiro, 2014) and have associated those mainly with hormonal changes (Weinstock et al., 1992; Weinstock, 2007).

In sum, we developed a new model of prenatal mild stress that presents emotional deficits that can now be used to explore in more detail how stress can imprint anatomical, molecular and functional changes in specific brain regions and lead to maladaptive behavior later in life.

## Author Contributions

AJR, CS-C and NS developed the concept and designed experiments. CS-C and BC performed stress protocol. CS-C, BC and SB performed and analyzed behavior. BC and CS-C performed molecular analysis. CS-C performed statistical analysis on all data. CS-C and AJR wrote the article. All authors discussed and revised the manuscript.

## Conflict of Interest Statement

The authors declare that the research was conducted in the absence of any commercial or financial relationships that could be construed as a potential conflict of interest.

## Supplementary Material

FIGURE S1 Prenatal stress (PS) animals of both genders present similar exploratory activity in the open field (OF). **(A)** Similar time spent in the center and periphery of the open arena between CTR and PS rats (males and females). **(B)** No differences in the distance traveled in the center and the periphery of the arena between groups (n_PS males_ = 9, n_CTR males_ = 9, n_PS females_ = 11, n_CTR females_ = 12). Error bars denote SEM.

FIGURE S2 Prenatal mild stress does not alter motivation for natural rewards or impulsivity. **(A)** Continuous reinforcement (CRF) and **(B)** fixed ratio (FR) trainings of male and female PS rats in the progressive ratio (PR) test, showing no differences in learning curves when compared to same-sex CTR group. **(C)** Breakpoint of PS and control animals, showing that PS exposure does not alter motivation to obtain food (n_PS males_ = 9, n_CTR males_ = 9, n_PS females_ = 12, n_CTR females_ = 12). **(D)** The number of food pellets earned during the PR session is similar between groups. **(E)** In the variable delay to signal (VDS), an impulsivity test, the number of premature responses of PS and CTR groups of both males and females is similar within each delay in the VDS test, indicating that PS does not change impulsivity (n_PS males_ = 9, n_CTR males_ = 9, n_PS females_ = 12, n_CTR females_ = 12). Error bars denote SEM.

TABLE S1 Number of pups per sex in each litter of stressed mothers (prenatal stress) and control mothers (control).
