# Supplementary figures and images for: Mild Prenatal Stress Causes Emotional and Brain Structural Modifications in Rats of Both Sexes
Source: Front Behav Neurosci. 2018 Jul 2;12:129. doi: 10.3389/fnbeh.2018.00129 (PMC6043801; doi:10.3389/fnbeh.2018.00129)

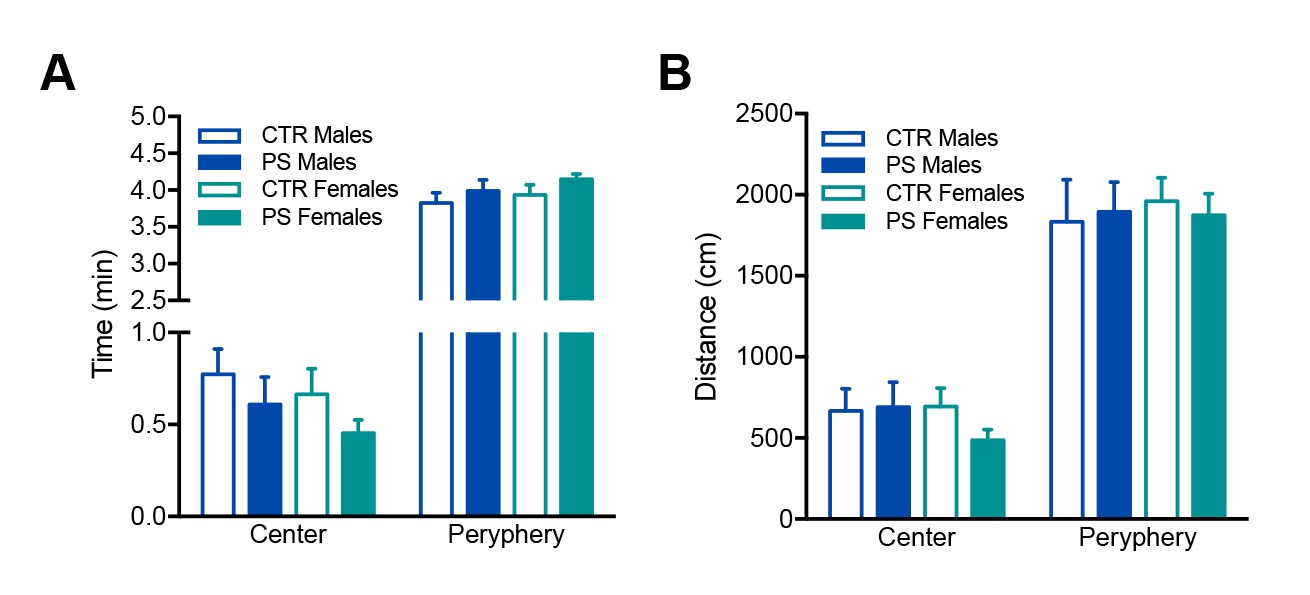

Supplement: FIGURE S1 — Prenatal stress (PS) animals of both genders present similar exploratory activity in the open field (OF). (A) Similar time spent in the center and periphery of the open arena between CTR and PS rats (males and females). (B) No differences in the distance traveled in the center and the periphery of the arena between groups (nPS males = 9, nCTR males = 9, nPS females = 11, nCTR females = 12). Error bars denote SEM. [file Image_1.TIF]

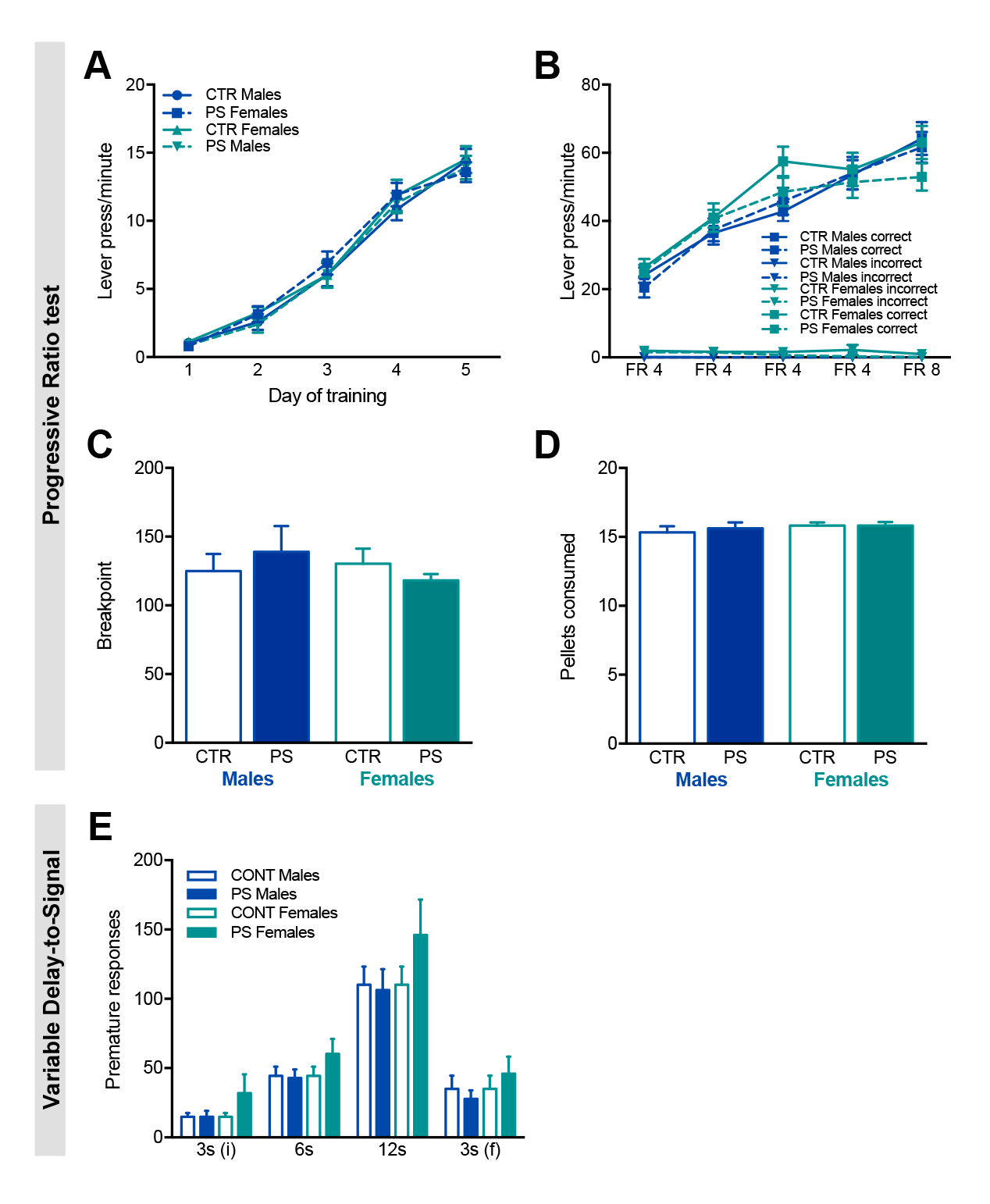

Supplement: FIGURE S2 — Prenatal mild stress does not alter motivation for natural rewards or impulsivity. (A) Continuous reinforcement (CRF) and (B) fixed ratio (FR) trainings of male and female PS rats in the progressive ratio (PR) test, showing no differences in learning curves when compared to same-sex CTR group. (C) Breakpoint of PS and control animals, showing that PS exposure does not alter motivation to obtain food (nPS males = 9, nCTR males = 9, nPS females = 12, nCTR females = 12). (D) The number of food pellets earned during the PR session is similar between groups. (E) In the variable delay to signal (VDS), an impulsivity test, the number of premature responses of PS and CTR groups of both males and females is similar within each delay in the VDS test, indicating that PS does not change impulsivity (nPS males = 9, nCTR males = 9, nPS females = 12, nCTR females = 12). Error bars denote SEM. [file Image_2.TIF]
