# Supplementary material for: Mild Prenatal Stress Causes Emotional and Brain Structural Modifications in Rats of Both Sexes
Source: Front Behav Neurosci. 2018 Jul 2;12:129. doi: 10.3389/fnbeh.2018.00129 (PMC6043801; doi:10.3389/fnbeh.2018.00129)
Supplement: TABLE S1 — Number of pups per sex in each litter of stressed mothers (prenatal stress) and control mothers (control). [file Table_1.DOCX]

| **Mother identification** | **Pups per litter** | |
| --- | --- | --- |
|  | **Males** | **Females** |
| Prenatal Stress 1 | 8 | 3 |
| Prenatal Stress 2 | 6 | 4 |
| Prenatal Stress 3 | 3 | 1 |
| Prenatal Stress 4 | 8 | 4 |
| Prenatal Stress 5 | 3 | 2 |
| Prenatal Stress 6 | 3 | 8 |
| Control 1 | 5 | 7 |
| Control 2 | 3 | 2 |
| Control 3 | 4 | 4 |
| Control 4 | 5 | 3 |
| Control 5 | 2 | 5 |
| Control 6 | 5 | 4 |

**Supplementary Table 1.** Number of pups per sex in each litter of stressed mothers (prenatal stress) and control mothers (control).
